# S-Nitrosoglutathione reduces oxidative injury and promotes mechanisms of neurorepair following traumatic brain injury in rats

**DOI:** 10.1186/1742-2094-8-78

**Published:** 2011-07-06

**Authors:** Mushfiquddin Khan, Harutoshi Sakakima, Tajinder S Dhammu, Anandakumar Shunmugavel, Yeong-Bin Im, Anne G Gilg, Avtar K Singh, Inderjit Singh

**Affiliations:** 1 Department of Pediatrics, Medical University of South Carolina, Charleston, SC 29425, USA; 2 Department of Pathology and Laboratory Medicine, Medical University of South Carolina, Charleston, SC 29425, USA; 3 Ralph H. Johnson V. A. Medical Center, Charleston, SC 29401, USA

## Abstract

**Background:**

Traumatic brain injury (TBI) induces primary and secondary damage in both the endothelium and the brain parenchyma, collectively termed the neurovascular unit. While neurons die quickly by necrosis, a vicious cycle of secondary injury in endothelial cells exacerbates the initial injury in the neurovascular unit following TBI. In activated endothelial cells, excessive superoxide reacts with nitric oxide (NO) to form peroxynitrite. Peroxynitrite has been implicated in blood brain barrier (BBB) leakage, altered metabolic function, and neurobehavioral impairment. S-nitrosoglutathione (GSNO), a nitrosylation-based signaling molecule, was reported not only to reduce brain levels of peroxynitrite and oxidative metabolites but also to improve neurological function in TBI, stroke, and spinal cord injury. Therefore, we investigated whether GSNO promotes the neurorepair process by reducing the levels of peroxynitrite and the degree of oxidative injury.

**Methods:**

TBI was induced by controlled cortical impact (CCI) in adult male rats. GSNO or 3-Morpholino-sydnonimine (SIN-1) (50 μg/kg body weight) was administered orally two hours following CCI. The same dose was repeated daily until endpoints. GSNO-treated (GSNO group) or SIN-1-treated (SIN-1 group) injured animals were compared with vehicle-treated injured animals (TBI group) and vehicle-treated sham-operated animals (Sham group) in terms of peroxynitrite, NO, glutathione (GSH), lipid peroxidation, blood brain barrier (BBB) leakage, edema, inflammation, tissue structure, axon/myelin integrity, and neurotrophic factors.

**Results:**

SIN-1 treatment of TBI increased whereas GSNO treatment decreased peroxynitrite, lipid peroxides/aldehydes, BBB leakage, inflammation and edema in a short-term treatment (4-48 hours). GSNO also reduced brain infarctions and enhanced the levels of NO and GSH. In a long-term treatment (14 days), GSNO protected axonal integrity, maintained myelin levels, promoted synaptic plasticity, and enhanced the expression of neurotrophic factors.

**Conclusion:**

Our findings indicate the participation of peroxynitrite in the pathobiology of TBI. GSNO treatment of TBI not only reduces peroxynitrite but also protects the integrity of the neurovascular unit, indicating that GSNO blunts the deleterious effects of peroxynitrite. A long-term treatment of TBI with the same low dose of GSNO promotes synaptic plasticity and enhances the expression of neurotrophic factors. These results support that GSNO reduces the levels of oxidative metabolites, protects the neurovascular unit, and promotes neurorepair mechanisms in TBI.

## Introduction

Neurobehavioral dysfunctions associated with traumatic brain injury (TBI) are the consequences of oxidative injury in the neurovascular unit that results in a damaging progression. These pathological events include continuous production of reactive oxidizing species and inflammation leading to disruption of the blood brain barrier (BBB), altered tissue homeostasis, axon/myelin loss, and cell death [1]. Previously, we reported the efficacy of S-nitrosoglutathione (GSNO) in TBI (short-term) [2], stroke [3,4] and spinal cord injury (SCI) [5]. In this report, we investigate the mechanisms of GSNO's action and test whether GSNO stimulates neurorepair processes in a clinically relevant two-week long TBI study.

GSNO, a modulator of cellular redox, is a physiological metabolite produced by the reaction of nitric oxide (NO) with glutathione (GSH) [6]. It is an efficient nitrosylating agent, and the mechanism of nitrosylation modulates protein functioning in health and disease [7,8]. Moreover, nitrosylation, like phosphorylation, functions as a signaling pathway and plays a major role in regulating several physiological and pathological processes. Under physiological conditions, GSNO and S-nitrosothiols are present in blood and brain [9-12]. The concentration of GSNO in adult rat brain tissue is estimated to be 6-8 μM, which is ~0.3 to 0.7% of the tissue GSH level [10]. A study on GSNO metabolism and its membrane crossing ability has been reported [13]. Using an *in vitro *BBB model, we have also reported that significant levels of GSNO cross the cellular membrane [3].

Pharmacologically, GSNO has been shown to protect the central nervous system (CNS) against excitotoxicity, inflammation, and reactive oxygen species (ROS) in a variety of injury conditions [14]. GSNO invokes its anti-inflammatory effects on post-injury events mainly through the down regulation of the expression of NF-κB, adhesion molecules, cytokines and inducible NOS (iNOS) [2,3,15-17]. It exerts its neuroprotective effects via reducing the neuronal apoptotic cell death and inhibiting the activity of caspase-3 [2,3]. GSNO shows its antioxidant action through the modulation of redox [18], such as increasing glutathione (GSH) [4] and decreasing peroxynitrite levels [19-21]. In acute CNS injury animal models, GSNO protects BBB, decreases edema, and reduces the expression of ICAM-1, ED1, and MMP-9 [2]. Furthermore, GSNO inhibits platelet activation, reduces embolization in humans [22-26], and inhibits inflammatory events in endothelial [17] and T cells [27]. Nitrosylation's protective effect in heart disease has also been observed [22,28,29]. Deficient nitrosylation seems to be a general mechanism of disease pathogenesis [30]. However, the potential of GSNO to stimulate the neurorepair process in human or animal brain trauma models, such as TBI, has not been investigated.

To investigate the GSNO-mediated mechanisms of neurorepair, we used a rat controlled cortical impact (CCI) model because approximately 40% of all TBIs are contusions [31]. The rat model of TBI using the focal CCI technique is recognized as physiologically relevant to human TBI [32]. It reproduces many of the features of human brain injuries including inflammation, BBB disruption, neuron loss, motor deficit, and compromised memory [33].

Presently, effective therapy for TBI patients is not available, mainly because of the limited understanding of the crosstalk between the multiple pathways involved in acute and chronic phases of the injury [1]. Delineation of these mechanisms is complicated due, in part, to excessive production of reactive oxygen/nitrogen species (ROS/RNS), not only in the brain cells but also in the endothelial cells. The involvement of ROS/RNS is supported by the observed reduced damage and improved brain functions afforded by free radical trapping agents and antioxidants in TBI [1,34-37] and stroke [38,39]. One of the highly deleterious ROS/RNS is peroxynitrite, a reaction product of a diffusion limited, instantaneous reaction between superoxide and NO. Peroxynitrite is documented to be highly injurious, and its reduction yields neuroprotection following experimental TBI and ischemia reperfusion (IR) injury [35,40-42]. Hence, 3-morpholino sydnonimine (SIN-1), a peroxynitrite forming agent, is deleterious in animal models of TBI [43] and stroke [4]. Treatment with GSNO has been shown to decrease the levels of peroxynitrite following brain injury [4,20]. GSNO also reduces inflammation, protects neurons from apoptotic cell death, and improves neurobehavioral functions following TBI [2], SCI [5], and IR [3,4]. However, the mechanisms through which GSNO reduces peroxynitrite, stimulates the neurorepair process, and improves neurobehavioral function are not clear.

In TBI, reduced NO bioavailability in the neurovascular unit is recognized to be the consequence of its conversion to peroxynitrite. Once formed, peroxynitrite can 'uncouple' endothelial NOS (eNOS) via oxidation and thus depleting the eNOS substrate L-arginine, the cofactor tetrahydrobiopterin (BH4), or both [44,45]. Excessive production of peroxynitrite can lead to endothelial cell activation and up regulation of ICAM-1 in such an oxidative microenvironment, thus compromising BBB integrity and contributing to edema [41]. Peroxynitrite is a highly reactive species for the nitration and inactivation of tyrosine in proteins in biological systems [46]. It also interacts with iron to form the most reactive hydroxyl radical [47]. Reducing iron or neutralizing ROS has been shown to be neuroprotective, indicating that iron and ROS play significant role in peroxynitrite-induced brain injury [48]. Antioxidant therapy down regulates neuroinflammation, stimulates the neurorepair process, and reduces neurological deficits following TBI [34,49].

In this study, we demonstrated that post-injury administration of GSNO reduced the oxidative injury in the neurovascular unit caused by peroxynitrite and its metabolites. The injury was associated with BBB leakage, neuroinflammation, edema, depletion of GSH and NO, and reduced expression of neurotrophic factors. Increased BBB leakage and edema, and an enhanced mRNA expression of ICAM-1 in the SIN-1 group compared to the TBI group indicate the involvement of peroxynitrite in BBB pathology. These results are supported by the increased formation of peroxynitrite and lipid peroxide/aldehydes and decreased levels of NO and GSH in the SIN-1 group. Moreover, a long-term (2-week) treatment with GSNO not only inhibited the loss of myelin and axons, but also enhanced the expression of neurotrophic factors. These findings indicate that GSNO not only down regulates TBI-induced neurovascular exacerbations but also stimulates the mechanisms of neurorepair.

## Methods

### Reagents

GSNO was purchased from World Precision Instruments (Sarasota, FL). 3-Morpholino-sydnonimine (SIN-1) was obtained from Cayman Chemical (Ann Arbor, MI). All other chemicals and reagents used were purchased from Sigma-Aldrich (St. Louis, MO), unless stated otherwise.

### Animals

Male Sprague-Dawley rats weighing 240-260 g (Harlan Laboratories, Wilmington, MA) were used in this study. All animal procedures were approved by the Medical University of South Carolina Animal Review Committee and received humane care in compliance with the Medical University of South Carolina's experimental guidelines and the National Research Council's criteria for humane care (*Guide for the Care and Use of Laboratory Animals*). Animals were not allowed to fast at any time in this study.

### Experimental design and administration of GSNO

The rats were randomly allocated into five groups: i) Sham (sham-operated control, n = 30); ii) TBI (vehicle-treated CCI, n = 40); iii) GSNO (GSNO-treated CCI, n = 40); iv) SIN-1 (SIN-1-treated CCI, n = 28); and v) Control (untreated normal, n = 13). Because GSNO-treated sham animals had no altered physiologic parameters (Table 1), including mean arterial blood pressure (MABP) and heart rate (HR), we did not include the Sham+GSNO group for further study. In the GSNO and SIN-1 groups, the rats were given freshly prepared GSNO (50 μg/kg body weight) and SIN-1 (50 μg/kg body weight), respectively, dissolved in sterile saline (~300 μl) by mouth using gavage needle at 2 hours after CCI. The rats in the TBI and the Sham groups were administered the same volume of saline. The same dose of GSNO and SIN-1 was repeated once every 24 hours by mouth until the endpoint (maximum 2 weeks) as described under each experiment. The dosages of both GSNO and SIN-1 used in this study were determined by a dose response curve ranging from 10 μg to 100 μg/kg body weight. The dose 50 μg/kg was found to be the most effective in reducing contusion volume measured at 7 days after CCI, as described in our previous TBI study [2]. The selected dosages of both GSNO and SIN-1 did not alter physiologic parameters including MABP, HR, and temperature, measured at 1 hour following GSNO or SIN-1 treatment (Table 1). Similar observations with low dose GSNO treatments were made in our TBI [2], SCI [5], and IR studies [3,4]. Blood pressure and HR were non-invasively measured by determining the tail blood volume with a volume pressure recording (VPR) sensor and an occlusion tail-cuff (CODA System, Kent Scientific, Torrington, CT).

**Table 1:** Physiologic parameters

	Sham						Injured (CCI)					
	**Untreated**		**GSNO-treated**		**SIN-1-treated**		**Untreated (TBI)**		**GSNO-treated (GSNO)**		**SIN-1 treated (SIN-1)**	

	**Basal**	**3 h**	**Basal**	**3 h**	**Basal**	**3 h**	**Basal**	**3 h CCI**	**Basal**	**3 h CCI**	**Basal**	**3 h CCI**

Rectal Temp (°C)	37.3 ± .3	37.2 ± .2	37.3 ± .2	36.9 ± .2	37.2 ± .1	36.8 ± .4	37.3 ± .2	36.6 ± .4	37.3 ± .3	36.7 ± .2	37.3 ± .2	36.6 ± .3

MABP (mm Hg)	123 ± 12	121 ± 11	122 ± 10	120 ± 12	122 ± 12	119 ± 13	121 ± 12	97 ± 14	116 ± 10	95 ± 9	118 ± 7	91 ± 8

HR (beat/min)	396 ± 32	394 ± 31	394 ± 32	404 ± 35	395 ± 31	408 ± 29	395 ± 30	405 ± 25	382 ± 27	410 ± 40	385 ± 24	415 ± 30

Measurements were performed before CCI (basal) and at 3 hours after CCI or 1 hour after drug administration (50 μg/kg body weight, oral). Data are presented as mean ± SD for n = 5 animals in each group. No significant differences were observed among untreated and treated groups of sham or injured animals. However, animals after CCI showed a trend of decreased MABP in the injured group. Basal, 30 min before CCI; Temp, temperature; MABP, mean arterial blood pressure; HR, heart rate.

### Controlled cortical impact (CCI) model of TBI

Surgical anesthesia was induced by ketamine (90 mg/kg body weight) and xylazine (10 mg/kg body weight) administered intraperitoneally (ip). Analgesic buprenorphine was administered pre-emptively to alleviate the pain following surgery. Utilizing aseptic techniques, a midline scalp incision was made, and the skin and fascia were reflected to expose the skull. A craniotomy was made in the right hemisphere, encompassing bregma and lambda, and between the sagittal suture and the coronal ridge with a handheld Michele trephine. The resulting bone flap was removed, and the craniotomy enlarged further with cranial rongeurs. This process did not cause rupture or significant bleeding. CCI injury was produced as previously described in the extensive literature [50-55]. A cortical contusion was produced on the exposed cortex using either a controlled impactor device described by Bilgen [56] and previously used in a rat model of SCI [5,57], or a TBI-0310 TBI Model system (Precision Systems and Instrumentation, LLC, Fairfax Station, VA) as described in our TBI study [2]. Briefly, the impacting shaft was extended, and the impact tip was centered and lowered over the craniotomy site until it touched the dura mater. Then, the rod was retracted and the impact tip was advanced farther to produce a brain injury of moderate severity (tip diameter, 4 mm; cortical contusion depth, 3 mm; impact velocity, 1.5 m/sec) [2]. The impact tip was wiped clean with sterile alcohol after each impact and cleaned/disinfected further with cidex after surgery. Core temperature was maintained at 37 ± 0.5°C with a heating pad during surgery and recorded with a rectal probe. Immediately after injury, the skin incision was closed with nylon sutures without replacing the bone flap. Lidocaine jelly (2%) was applied to the lesion site to minimize any possible infection/discomfort.

### Evaluation of BBB disruption by Evan's blue (EB) extravasation

BBB leakage was assessed as described [2] by the method of Weismann and Stewart [58] with slight modification. The rats received 100 μl of a 5% solution of EB in saline administered intravenously 4 hours following CCI. At 48 hours, cardiac perfusion was performed under deep anesthesia with 200 ml of saline to clear the cerebral circulation of EB. The brain was removed, sliced, and photographed. The two hemispheres were isolated and mechanically homogenized in 750 μl of N, N-dimethylformamide (DMF). The suspension obtained was kept at room temperature in the dark for 72 hr. It was centrifuged at 10,000 × g for 25 minutes, and the supernatant was spectrofluorimetrically analyzed (λ_ex _620 nm, λ_em _680 nm) to determine EB content.

### Measurement of Edema (brain water content)

At 24 h following CCI, animals were euthanized to determine brain water content (edema) as described earlier [2,59]. The cortices, excluding the cerebellum, were quickly removed, and the contralateral and ipsilateral hemispheres separately weighed. Each hemisphere was dried at 60°C for 72 hours, and the dry weight was determined. Water content was calculated in ipsilateral hemisphere as: water content (%) = (wet weight - dry weight)/wet weight × 100.

### Dot blot analysis

Dot blot for the expression of 3-NT and β-actin in the traumatic penumbra region was performed as described by Neumann et al [60]. In brief, PVDF membranes were hydrated in TBS and then placed in a Bio-Dot microfiltration. Wells were washed with 100 μl of TBS under a partial vacuum. Wells were loaded with 100 μg (75 μl) protein each. The membrane was washed in TBS and blocked 30 min in blotto (TBS, 5.0% non-fat dry milk, 0.05% Tween 20) at room temperature. The membrane was then incubated with monoclonal anti-nitrotyrosine antibody (Abcam, Cambridge, MA; dilution 1:1000), or anti-β-actin antibody in blotto for 3 h at room temperature. The blot was washed in TTBS (TBS, 0.05% Tween 20). The blot was then incubated with a secondary antibody for 45 min and washed in TTBS 3 times for 5 minutes. The blot was developed as described elsewhere for Western blot [2]. The bands were quantitated using a GS-800 calibrated densitometer from Bio-Rad Laboratories (Hercules, CA, USA). Protein concentrations were determined using protein assay dye from Bio-Rad Laboratories (Hercules, CA).

### Histopathology, inflammation, and demyelination

Histological evaluation was done on paraformaldehyde-fixed, paraffin-embedded sections (4 μM) of brain. Sections were stained with hemotoxylin and eosin (H&E), luxol fast blue (LFB), and Bielshowsky silver impregnation to assess inflammation, demyelination, and axonal pathology, respectively. H&E and LFB staining were performed as described previously from our laboratory [61]. For Bielschowsky silver immunostaining, slides were placed in 20% silver nitrate in the dark, and then sodium hydroxide and sodium thiosulfate were added to the slides in turn as described earlier [62]. The histological analysis was performed by an investigator who was blinded to the experimental groups.

### Immunohistochemistry (IHC)

Paraffin-embedded sections from the formalin-fixed brain tissues were stained for 3-NT, 4-HNE, ferritin, von Willebrand factor (vWF), platelet endothelial cell adhesion molecule 1 (PECAM-1), NeuN, synaptophysin, BDNF, and TrkB as described previously [2]. In brief, the brain tissue sections were deparaffinized, sequentially rehydrated in graded alcohol, and then immersed in PBS (pH 7.4). Slides were then microwaved in antigen unmasking solution (Vector Labs), cooled, and washed 3 times for 2 minutes in PBS. Sections were incubated overnight with antibodies of 3-NT, NeuN, synaptophysin, BDNF, TrkB, ferritin (abcam, Cambridge, MA), 4-HNE (A. G. Scientific, San Diego, CA), vWF, and PECAM-1 (Santa Cruz Biotechnology, Santa Cruz, CA). They were then rinsed 3 times for 5 minutes in PBS containing 0.1% Tween-20. Secondary anti-rabbit and mouse IgG, conjugated with Alexa Fluor 568 and 488, respectively, were incubated on slides for 60 minutes. BDNF sections were allowed to react with anti-mouse IgG conjugated to a peroxidase-labeled dextran polymer (for 60 min) and then developed with diaminobenzidine substrate for 10 min at room temperature. All sections were examined for immunoreactivity in the traumatic penumbral area using an Olympus microscope equipped for epifluorescence and DP Controller software. Figures were compiled in Adobe Photoshop software.

For double labeling, sections were incubated first with an antibody of 3-NT followed by specific cell marker antibodies (vWF, NeuN, or PECAM-1). For immunofluorescent double-labeling, immune complexes were visualized with Texas Red conjugated anti-rabbit IgG (1:100, Vector Labs) and FITC conjugated anti-mouse IgG (1:100, Vector Labs). Rabbit polyclonal IgG was used as a control primary antibody. In another set of experiments, sections were also incubated with FITC or Texas Red conjugated IgG without the primary antibody as a negative control to confirm the specificity of the primary antibody. Slides were examined for immunofluorescence using an Olympus microscope equipped with an epifluorescence filter and Adobe Photoshop software [3].

Semi-quantitative cell counting was performed in three equi-distant sections (10 sections apart) from each brain and expressed as an average number of immunopositive cells per section. The positive cells were counted on each section at 400 × magnification (one visual field = 0.69 mm^2^) using an Olympus microscope equipped with epifluorescence and DP Controller software. Images were captured and processed using Adobe Photoshop software.

### Real-time polymerase chain reaction (RTPCR)

Total RNA from the traumatic penumbra was isolated with TRIZOL (Invitrogen, Carlsbad, CA) according to the manufacturer's protocol. RTPCR was conducted using Bio Rad iCycler (iCycler iQ Multi-Color Real Time PCR Detection System; Bio Rad, Hercules, California). Single stranded cDNA was synthesized from total RNA isolated from sham, TBI, GSNO, and SIN-1-treated brains at 24 h and 14 days following TBI using the superscript pre-amplification system for first strand cDNA synthesis (Invitrogen, Carlsbad, CA) as described earlier [63]. The primer sets used were designed by using free software available at http://www.idt.com and were purchased from Integrated DNA technologies (IDT, Coralville, IA). The primer sequences were as follows: for intercellular adhesion molecule 1 (ICAM-1), forward primer: 5'-GTCCTTCACACTGAATGCCAGC-3'; reverse primer: 5'-TTAAACAGGAACTTTCCCGCCACC-3', β-actin, forward primer: 5'-AGCTGTGCTATGTTGCCCTAGACT-3'; reverse primer: 5'-ACCGCTCATTGCCGATAGTGATGA-3'. IQ™ SYBR Green Supermix was purchased from Bio-Rad (BIO-RAD Laboratories, Hercules, CA). Thermal cycling conditions were as follows: activation of iTaq TM DNA polymerase at 95°C for 10 min, followed by 40 cycles of amplification at 95°C for 30 sec and 55-57.5°C for 30 - 60 seconds. The detection threshold was set above the mean base line fluorescence determined by the first 20 cycles. Amplification reaction in which the fluorescence increased above the threshold was defined as positive. A standard curve for each template was generated using serial dilution of the template (cDNA). The quantities of target gene expression were normalized to the corresponding β-actin mRNA quantities in test samples.

### Evaluation of infarction using TTC staining

To evaluate infarct, a 2, 3, 5-triphenyltetrazolium chloride (TTC) staining technique was used followed by image acquisition by computer. Briefly, after an overdose of pentobarbital, the rats were killed by decapitation at 48 h of CCI. The brains were quickly removed and placed in ice-cold saline for 5 min. Six serial sections from each brain were cut at 2-mm intervals from the frontal pole using rodent brain matrix (ASI Instrument Inc., Warren, MI). The sections were incubated in 2% TTC saline solution for 15 min at 37°C. The stained brain sections were stored in 10% formalin and refrigerated at 4°C for further processing and storage. Coronal sections (2 mm) were placed on a flat bed color scanner (HP scan jet 5400 C) connected to a computer. The infarct area, outlined in white, was acquired by image-analysis software (Adobe Photoshop software) and measured by NIH image software.

### Estimation of peroxynitrite in plasma

The plasma samples collected at 4 h of CCI were quantified for nitrotyrosine by BIOXYTECH^® ^Nitrotyrosine -EIA kit from Oxis Research (Portland, OR) per the manufacturer's instructions and as described previously [4]. Briefly, 100 μl of samples (diluted 10 times in the dilution buffer) were added to each well of a precoated plate for 1 h at room temperature. After four washes with wash buffer, nitrotyrosine antibody was added to each well and incubated at room temperature for 1 h. Subsequently, streptavidin peroxidase was added and the plate incubated for 1 h at room temperature. Tetramethylbenzidine (TMB) substrate was added, and the plate was incubated in the dark at room temperature to complete the reaction. The optical density of the assay samples was measured spectrophotometrically at 450 nm using SpectraMax 190 (Molecular Devices, San Diego, CA). A standard curve derived from nitrotyrosine standards (provided with the kit) was used to calculate the nitrotyrosine concentrations.

### Measurement of NO in plasma

The plasma samples collected at 4 h of CCI were quantified for the levels of NO using a Nitric Oxide fluorometric assay kit from BioVision (Exton, PA). Briefly, 2 μl plasma was used to assay NO (measured as the concentration of nitrite and nitrate as an index of NO levels) by 2, 3-diaminonaphthalene (DAN) reaction, using a fluorometer (Ex. 360 nm and Em. 450 nm) per manufacturer's instructions. Nitrate reductase was used to convert nitrate in nitrite. Nitrite concentrations (NO levels) were calculated from a standard curve derived from sodium nitrite (provided with the kit).

### Estimation of lipid peroxidation products in plasma

The plasma samples (100 μl/assay) collected at 4 h of CCI were quantified for thiobarbituric acid reactive substances (TBARS) using a TBARS assay kit from ZeptoMetrix Corporation (Buffalo, NY) per manufacturer's instructions and as described previously [4]. TBARS were quantitated spectrophotometrically at 532 nm. A standard curve derived from malondialdehyde (MDA) was used to calculate the level of TBARS.

### Measurement of the ratio GSH:GSSG

The levels of total glutathione (GSH and GSSG) were measured at 24 h in the penumbra using a BIOXYTECH^® ^GSH/GSSG-412™ colorimetric assay kit from Oxis Research (Portland, OR). The kit uses 5, 5'-dithiobis-2-nitrobenzoic acid (DTNB) and glutathione reductase as described by Tietze [64] with some modification. It uses 1-methyl-2-vinylpyridinium trifluoromethanesulfonate (M2VP) instead of N-ethylmaleimide (NEM). Brain tissues harvested at 24 h of CCI were minced and homogenized (g/10 ml) in 5% metaphosphoric acid. The procedure was followed per manufacturer's instructions and has been used in our previous studies [4,65]. The levels were quantitated as μM GSH or GSSG based on standards supplied with the kit. Total GSH (TGSH) levels were the sum of GSH and GSSH.

#### Statistical evaluation

Statistical analysis was performed as described [66] using software Graphpad Prism 3.0. Unless otherwise stated, all the values are expressed as mean ± SD of n determinations or as mentioned. The results from biochemical and animal behavior studies were examined by unpaired Student *t*-test. Multiple comparisons were performed using the Kruskal-Wallis test, or using ANOVA followed by the Bonferroni test as appropriate. A p value less than 0.05 was considered significant.

## Results

### Peroxynitrite is formed in and around the vessels after TBI, and GSNO treatment reduces the expression of peroxynitrite

**Figure 1:**
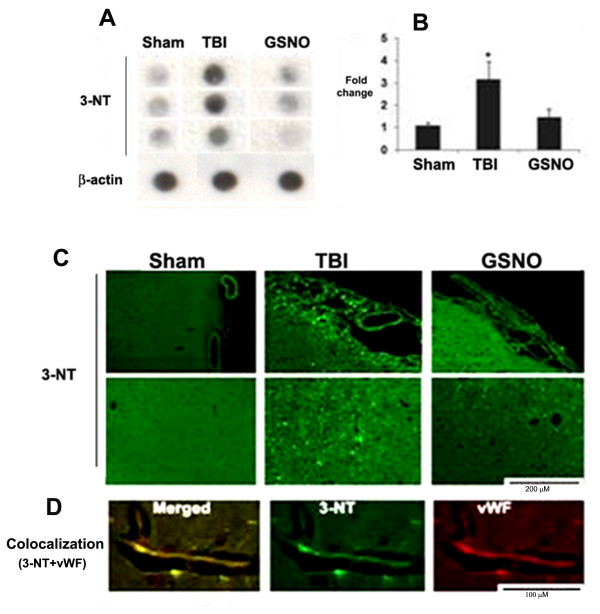
**Effect of GSNO on the expression of 3-NT determined by dot blot and IHC at 4 h after TBI and colocalization of 3-NT with vWF**. Animals were treated with GSNO (50 μg/kg body weight) at 2 h after TBI. Dot blot (A) and its densitometric analysis (B), performed at 4 h after TBI, show the accumulation of 3-NT in the traumatic penumbra region. The expression of 3-NT was significantly reduced in the GSNO group. Photomicrographs of IHC of 3-NT (C), determined at 4 h after TBI, show the enhanced expression of 3-NT in and around vessels. Treatment with GSNO reduced the TBI-mediated increased expression of 3-NT. Colocalization of 3-NT with an endothelial cell marker vWF as yellowish fluorescence (D) indicates that 3-NT was expressed in endothelial cells. Dot blots (A, B) and photomicrographs (C, D) are representative of n = 3 in each group. Densitometry results (B) are expressed as fold change, and data are presented mean ± SD. *p < 0.05 vs. Sham and GSNO.

**Figure 2:**
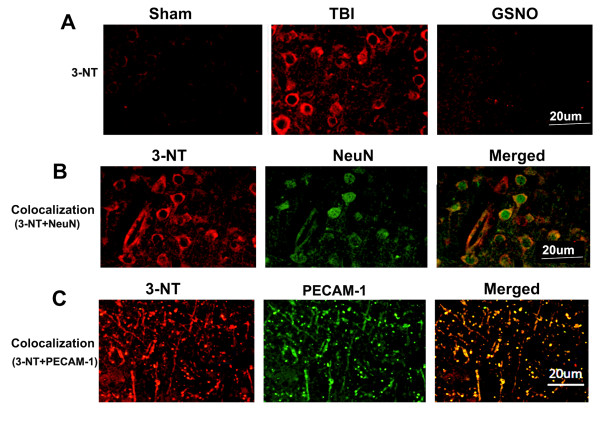
**Effect of GSNO on reduction of the expression of 3-NT at 14^th ^day after TBI**. Photomicrographs of IHC show enhanced reaction of 3-NT (red color) in the traumatic penumbra region of TBI compared to GSNO group. Sham brain does not show 3-NT-positive cells. Colocalization (yellowish, merged) of 3-NT (red color) with either neuronal marker NeuN (green) (B) or endothelial cell marker PECAM-1 (green) (C) indicates that both neurons and endothelial cells have significantly increased expression of 3-NT even after 14 days of TBI. Photomicrographs are representative of n = 3 in each group.

We have previously reported that GSNO protects not only the neurovascular unit after TBI and stroke [2-4] but also reduces the levels of peroxynitrite in plasma following IR [4]. Therefore, we investigated whether GSNO also reduces the levels of peroxynitrite, measured as the expression of 3-nitrotyrosine (3-NT) in TBI. Because peroxynitrite is unstable, its detection through 3-NT expression is an index of the levels of peroxynitrite [67]. The expression of 3-NT was measured at 4 h following TBI in the traumatic penumbra region using dot blot [60] and IHC. GSNO (50 μg/kg body weight) treatment significantly reduced the TBI-mediated increased expression of 3-NT as shown by dot blotting (Figure 1A) and its densitometry (Figure 1B). A similar decrease of the expression of 3-NT by GSNO was also observed using IHC (Figure 1C). The expression was remarkable in and around vessels as indicated by its colocalization with vWF, an endothelial cell marker (Figure 1D). However, the expression of 3-NT at 4 h was not observed in other cell types, including neurons (data not shown). In a long-term study (2-week), the expression of 3-NT was found to be remarkably increased in the traumatic penumbra (Figure 2A). After two weeks, the expression of 3-NT was observed in both neurons (Figure 2B) and endothelial cells (Figure 2C) as indicated by colocalization of 3-NT with NeuN (neuronal cell marker) and PECAM-1 (endothelial cell marker), respectively. Treatment with GSNO decreased the expression of 3-NT (Figure 2A). In long-term study, PECAM-1 was used as an endothelial cell marker because it detects mature endothelial cells.

### GSNO decreases and SIN-1 increases the levels of 3-NT in plasma following TBI

**Figure 3:**
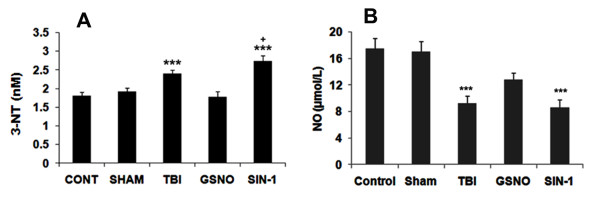
**Effect of GSNO and SIN-1 on levels of 3-NT and NO in plasma at 4 h after TBI**. Levels of 3-NT (A) and NO (B) were measured in plasma using colorimetric and fluorometric assay kits, respectively. While GSNO treatment of TBI decreased, the SIN-1treatment increased the levels of 3-NT (A). GSNO treatment also increased the levels of NO, whereas SIN-1 treatment did not alter the levels of NO in the injured animals. Results are expressed as nM for 3-NT and μmol/L for NO. Data are presented as mean ± SD of triplicate determinations from five different experiments. ***p < 0.001 vs. Control, Sham, GSNO, +p < 0.05 vs. TBI.

To test whether GSNO decreases and SIN-1 (a peroxynitrite forming agent) increases TBI-mediated accumulation of peroxynitrite in plasma and whether the plasma levels correlate with the tissue levels, we determined 3-NT levels in plasma at 4 h in TBI, GSNO, and SIN-1 groups. The selected dose (50 μg/kg body weight) of GSNO and SIN-1 did not alter any of the physiologic parameters, including rectal temperature, MABP, and HR (Table 1). A similar dose of GSNO provided the neurovascular protection as reported previously [2]. While GSNO treatment significantly reduced the TBI-mediated levels of 3-NT, SIN-1 significantly increased the levels (Figure 3A). SIN-1 treatment also significantly increased the levels of 3-NT compared with TBI. These results agree with a published report showing increased levels of 3-NT after SIN-1 treatment [42]. Furthermore, the levels of 3-NT in plasma (Figure 3A) had a correlation with the observed increased expression of 3-NT in the traumatic penumbra (Figure 1).

### GSNO increases and SIN-1 decreases the levels of NO in plasma following TBI

Peroxynitrite is formed instantaneously through a diffusion limited reaction between superoxide and NO, resulting in decreased levels of NO [67]. We determined whether increased levels of peroxynitrite corresponded to the decreased levels of NO in TBI and SIN-1-treated plasma samples at 4 h following TBI. While GSNO treatment significantly increased the TBI-mediated decreased levels of NO, SIN-1 significantly decreased the levels (Figure 3B). SIN-1 treatment also significantly decreased the levels of NO compared with the TBI plasma. Expectedly, the levels of NO in plasma had an inverse relationship with the levels of 3-NT in plasma (Figure 3A-B).

### GSNO decreases and SIN-1 increases the levels of TBARS in plasma, and GSNO also reduces the expression of 4-HNE and ferritin in the traumatic penumbra region following TBI

**Figure 4:**
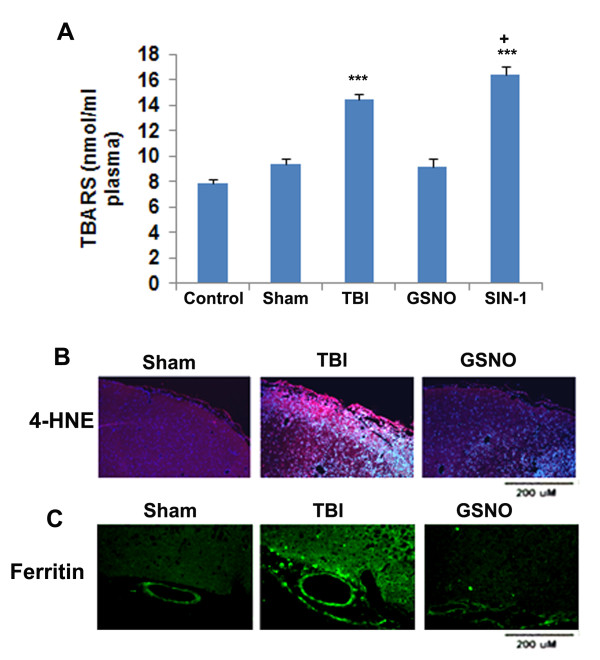
**Effect of GSNO and SIN-1 on levels of TBARS in plasma and on the expression of 4-HNE and ferritin in the traumatic penumbra region at 4 h after TBI**. Levels of TBARS were measured in plasma using TBARS assay kit. While GSNO treatment of TBI decreased, the SIN-1 treatment increased the levels of TBARS (A). TBARS results are expressed as nmol/ml plasma, and data are presented as mean ± SD of triplicate determinations from five different experiments. ***p < 0.001 vs. Control, Sham, GSNO, +p < 0.05 vs. TBI. The expression of 4-HNE (B) and ferritin (C) was determined using IHC. Photomicrographs show enhanced reaction of both 4-HNE and ferritin in the traumatic penumbra compared to GSNO group. Sham brain does not show significant number of 4-HNE or ferritin-positive cells. Photomicrographs are representative of n = 3 in each group.

In addition to nitration and inactivation of the tyrosine residue of a protein forming 3-NT, peroxynitrite is implicated in tissue injury via lipid peroxidation [47]. Peroxynitrite forms a highly reactive hydroxyl radical in the presence of free iron. Ferritin is a key iron storage protein, and its expression is increased in the presence of free iron [68]. Therefore, we determined the levels of the lipid peroxidation products TBARS (Figure 4A) in plasma and the expression of 4-HNE (Figure 4B) in the traumatic penumbra at 4 h following TBI. We also measured the expression of ferritin (Figure 4C), supporting that decomposition of peroxynitrite due to the presence of excessive free iron may be responsible for the increased levels of lipid peroxidation products. Like its effect on 3-NT (Figure 3A), the GSNO treatment significantly reduced the TBI-mediated accumulation of TBARS in plasma (Figure 4A). Contrary to GSNO's inhibitory effect on TBARS, SIN-1 treatment increased the levels of TBARS (Figure 4A). Remarkably, SIN-1 treatment increased the levels of TBARS even further than TBI (p < 0.05), indicating peroxynitrite participation in lipid peroxidation. Similar to the effect of GSNO on TBARS (Figure 4A) and 3-NT (Figure 3A) in plasma, the treatment also reduced the expression of 4-HNE in the traumatic penumbra (Figure 4B). GSNO treatment of TBI also reduced the expression of ferritin (Figure 4C), indicating that the GSNO treatment down regulated the overall oxidative burden.

### GSNO decreases and SIN-1 increases BBB leakage and edema following TBI

**Figure 5:**
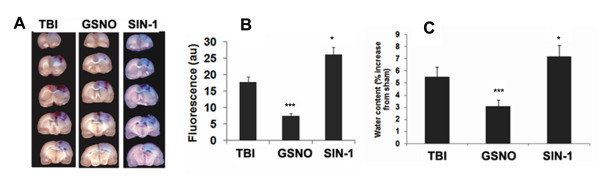
**Effect of GSNO and SIN-1 on BBB leakage and edema**. Photographs showing Evan's blue (EB) extravasations in five coronal sections of brain starting at 4 h after TBI. Animals were sacrificed at 48 h, the brain was sectioned and photographed (A) and the intensity of EB (B) was determined by spectrofluorometric estimation. EB extravasations were not observed in sham brain, hence the photographs of sham brain are not shown. Edema (C) was measured at 24 h after TBI. While GSNO treatment of TBI decreased, SIN-1treatment increased both the Evan's blue extravasations and edema. Data are expressed as mean ± SD from five different experiments. ***p < 0.001 vs. TBI and SIN-1, *p < 0.05 vs. TBI.

BBB disruption and edema indicate pathology associated with endothelial cell activation and endothelial dysfunction [69]. Peroxynitrite, through 3-NT formation, has been implicated in cerebral vascular dysfunction and the consequent BBB disruption [41,70,71]. In contrast, the nitrosylating agent GSNO has been shown to reduce BBB leakage and edema [2], and to provide protection to neurons and vessels [4,18,20]. To compare the neurovascular protective action of GSNO/nitrosylation and the deleterious action of SIN-1/3-NT, we examined the effect of GSNO and SIN-1 on BBB leakage and edema following TBI. Extravasations of Evan's blue dye into the brain of TBI were higher than in the GSNO brain (Figure 5A-B). Unlike the effect of GSNO, treatment with SIN-1 increased Evan's blue extravasations. Moreover, the SIN-1 mediated extravasations were greater than TBI, indicating that peroxynitrite causes neurovascular oxidative injury. A study of edema at 24 h after TBI showed increased levels of water content in the TBI brain (Figure 5C). While the treatment with GSNO decreased the water content, SIN-1 treatment increased it, again beyond the levels in the TBI brain. This comparative study of GSNO and SIN-1 on BBB integrity indicates that the nitrosylating agent GSNO provides neurovascular protection and the peroxynitrite-producing agent SIN-1 worsens injury.

### GSNO down regulates mRNA expression of ICAM-1in the traumatic penumbra region following TBI

**Figure 6:**
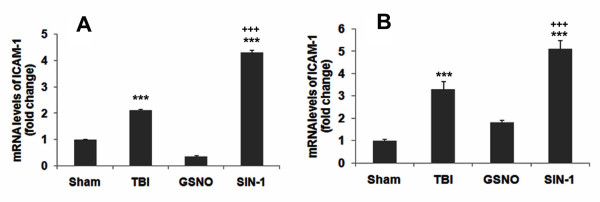
**Effect of GSNO and SIN-1on reduction of mRNA expression of ICAM-1 in brain at 24 h and 14^th ^day after TBI**. RNA was isolated from Sham, TBI, GSNO and SIN-1 treated traumatic penumbra region at 24 h (A) and 14^th ^day (B) after TBI. Levels of ICAM-1 were determined by RT-PCR and normalized with β-actin. While GSNO treatment decreased, SIN-1 treatment increased ICAM-1 levels at both 24 h (A) and 14 days (B). Data are presented as mean ± SD (fold change) of duplicate determinations from three different experiments. ***p < 0.001 vs. Sham and GSNO, +++p < 0.001 vs. TBI.

ICAM-1 mediates endothelial dysfunction that facilitates BBB leakage, resulting in the transmigration of blood-derived immune cells in the brain and thereby maintaining a persistent inflammation following TBI [72]. Therefore, we determined whether TBI-mediated increased mRNA expression of ICAM-1 is decreased at both short- and long-term GSNO treatment, thus reducing inflammation and stimulating the mechanisms of neurorepair. The traumatic penumbra region had increased mRNA levels of ICAM-1 at both 24 h (Figure 6A) and 14 days (Figure 6B) following TBI. GSNO treatment prevented the increase in mRNA levels of ICAM-1, whereas the treatment with SIN-1 increased the levels at both 24 h and 14 days (Figure 6). The sham-operated brain had no significant mRNA levels of ICAM-1.

### GSNO reduces brain infarctions following TBI

**Figure 7:**
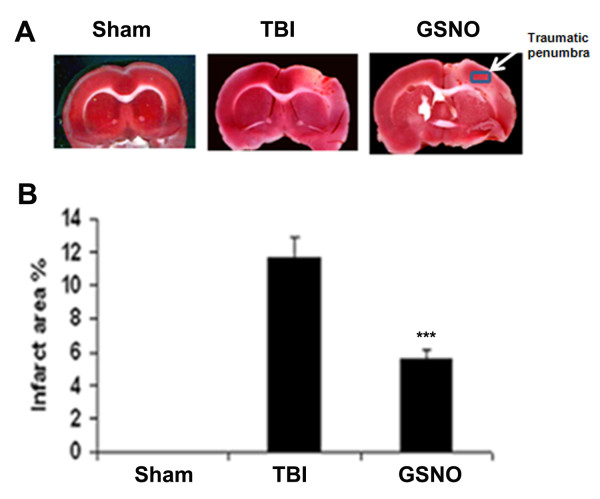
**Effect of GSNO on reduction of infarction after TBI**. Representative TTC-stained brain section (#3 out of the six consecutive sections from cranial to caudate region) corresponding to the largest infarction (A) and infarct area (B) from each group. Brain sections (2 mm thick) were stained with TTC at 48 h after CCI to show the area of infarctions. The infarctions were not observed in the sham group. The area under the rectangle on GSNO section was used for IHC and histology studies. Data are presented as means ± SD from three different experiments. *** p < 0.001 vs. TBI.

Directly related to overall brain injury, measurement of brain infarctions is a standard method to evaluate ischemic injury after stroke [73]. Previous study shows that treatment with GSNO reduces infarctions following IR [3] and inhibits apoptotic cell death following TBI [2]. To evaluate the effect of GSNO on brain infarctions in the TBI brain, we performed TTC staining (Figure 7A) and measured the infarct area (Figure 7B). The infarctions were significantly reduced after treatment with GSNO (Figure 7A-B). Measuring infarctions also served the purpose of locating the traumatic penumbra as shown by a rectangle on the GSNO section (Figure 7A). The area under the rectangle was used in IHC and histological studies.

### GSNO increases and SIN-1 decreases the levels of both reduced glutathione (GSH) and total glutathione (TGSH; GSH+GSSG) in the traumatic penumbra region following TBI

**Figure 8:**
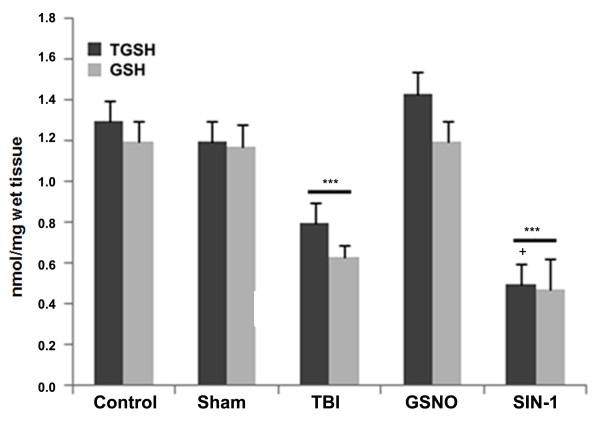
**Effect of GSNO and SIN-1 on levels of reduced glutathione (GSH) and total glutathione (TGSH; GSH+GSSG) in the traumatic penumbra at 24 h after TBI**. Levels of GSH and GSSG were measured in plasma using a colorimetric assay kit. Results are expressed as nmol/mg wet tissue, and data are presented as mean ± SD of triplicate determinations from five different experiments. ***p < 0.001 vs. Control, Sham, GSNO, +p < 0.05 vs. TBI.

GSH is an endogenous antioxidant that protects endothelial function and improves NO bioavailability in the neurovascular unit following acute injury [74]. With NO and oxygen, GSH forms GSNO [75], and GSNO is metabolized into GSSG (oxidized GSH) during nitrosylation *in vivo *[6,10]. We tested whether GSNO, a strong antioxidant against peroxynitrite [20], increased, and whether SIN-1, a strong oxidant [43], decreased the levels of GSH and TGSH. The TBI brain had significantly lower levels of both GSH and TGSH than control, sham, and GSNO brains measured at 24 h following CCI (Figure 8). However, the SIN-1-treated brain had significantly reduced levels of both compared to even the TBI brain (Figure 8). These results show that peroxynitrite causes its deleterious effects, at least in part, by reducing the levels of GSH.

### GSNO protects the integrity of brain tissue and axons, and enhances the levels of myelin following TBI

**Figure 9:**
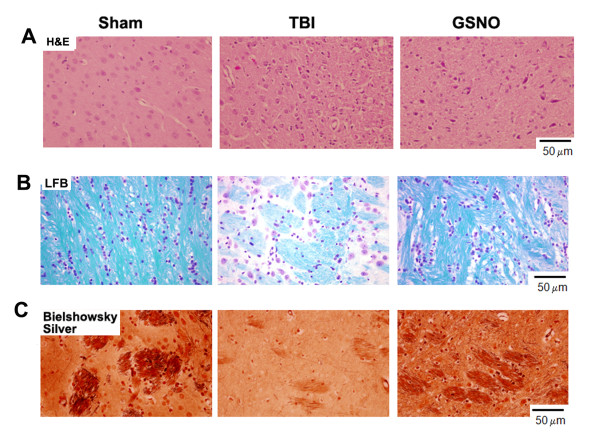
**Effect of GSNO on reduction of inflammation, demyelination and axonal loss at 14^th ^day after TBI**. IHC photomicrographs of H&E staining (A) show enhanced inflammatory infiltration in the traumatic penumbra region of TBI group compared with GSNO group. Sham group does not show infiltration. LFB (B) and Bielschowsky silver (C) stainings show loss of myelin and axons respectively in the traumatic penumbra region. Treatment with GSNO reduced the loss of myelin and damage to axons. The sham brain does not show loss of myelin or axons. Photomicrographs are representative of n = 3 in each group.

To examine whether GSNO treatment of TBI maintains tissue structure and aids the neurorepair process, tissue histology using H&E (Figure 9A), myelin content using LFB (Figure 9B), and axonal integrity using Bielschowsky silver (Figure 9C) stainings were performed in the fixed sections at 14 days after TBI. H&E staining shows that TBI-mediated tissue deformation and infiltration of immune cells were blocked by GSNO treatment. Furthermore, myelin levels were reduced following TBI (Figure 9B). GSNO treatment also reduced the loss of myelin, indicating an induction of the neurorepair process. Bielschowsky silver staining is a marker of axonal integrity, which was also reduced in TBI (Figure 9C). The treatment with GSNO protected axonal integrity, as indicated by the enhanced staining of Bielschowsky silver in the GSNO brain.

### GSNO increases the expression of BDNF, TrkB, and synaptophysin in the traumatic penumbra region following TBI

**Figure 10:**
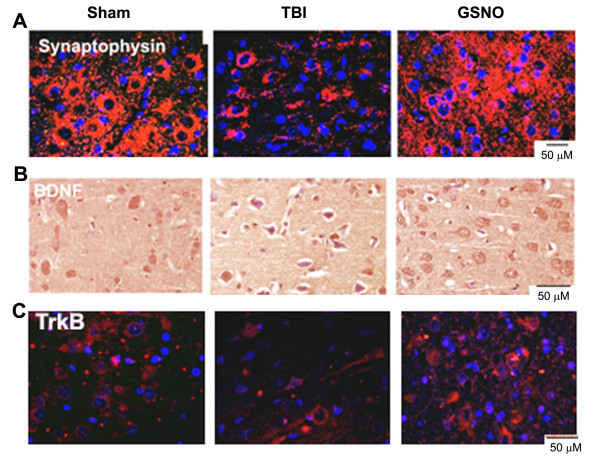
**Effect of GSNO on enhanced expression of synaptophysin, BDNF, and TrkB at 14^th ^day after TBI**. IHC photomicrographs show reduced expression of synaptophysin (A, red fluorescence), BDNF (B, brown diaminobenzidine staining), and TrkB (C, red fluorescence) in the traumatic penumbra region of the TBI group. Sham and GSNO groups show enhanced expression of synaptophysin, BDNF and TrkB compared to the TBI group. Photomicrographs are representative of n = 3 in each group.

Synaptic plasticity and neurotrophic factors (expression of synaptophysin, BDNF, TrkB) are involved in neurorepair activity, leading to functional recovery following acute brain injury [76-78]. While the expression of synaptophysin is related with synaptogenesis, the expression of BDNF and its receptor TrkB supports neuron survival and axon growth after neuronal injury [76]. We examined whether GSNO induces the neurotrophic factors and enhances their expression, thereby contributing to the overall neurorepair process. The expression was determined in the fixed sections at 14 days after TBI in the traumatic penumbra. The expression of synaptophysin (Figure 10A), BDNF (Figure 10B) and TrkB (Figure 10C) was remarkably decreased in the TBI brain compared to sham. The GSNO-treated brains showed intense expression of synaptophysin, BDNF, and TrkB compared to the TBI brains (Figure 10 A-C).

## Discussion

Following TBI, 3-NT (peroxynitrite) is formed early in and around the vessels (Figure 1) and in plasma (Figure 3A). The expression of 3-NT remains increased in neurons as well as in endothelial cells even two weeks after TBI (Figure 2). The increased 3-NT levels (Figures 1, 2, 3) correlated with the levels of lipid peroxidation products in both brain and plasma (Figure 4). While GSNO treatment of TBI animals significantly reduces the levels of 3-NT and lipid peroxidation products (Figures 1, 2, 3, 4), SIN-1 (a peroxynitrite forming agent) treatment significantly increases the levels of peroxynitrite (Figure 3A) and TBARS (Figure 4) in plasma compared to the TBI group. Furthermore, SIN-1 treatment of TBI increases BBB leakage (Figure 5A), edema (Figure 5C), and the expression of ICAM-1 (Figure 6), supporting the involvement of peroxynitrite in TBI-induced neurovascular pathology. Also, SIN-1 treatment of TBI remarkably decreases and GSNO significantly increases the levels of NO (Figure 3B) and GSH compared to TBI (Figure 8), indicating an association of antioxidant activity with GSNO. This antioxidant activity of GSNO might be responsible, at least in part, for its neurovascular protective ability. In the long-term two-week study, GSNO treatment of TBI stimulated the neurorepair mechanisms by blocking the loss of myelin/axons (Figure 9) and enhancing the expression of both synaptophysin and neurotrophic factors such as BDNF and its high affinity receptor TrkB (Figure 10).

The deleterious role of peroxynitrite in both IR and TBI is accepted because the scavenging of peroxynitrite has resulted in neurovascular protection [41,42,67,71,79]. However, reducing peroxynitrite for therapeutic purposes by GSNO has an advantage over reported metal-based synthetic scavengers [42,80] because GSNO is an endogenous molecule of the human body and it is metabolized ultimately into beneficial -SNO/NO and GSH [18]. In the traumatized brain, NO bioavailability decreases early due to its conversion to peroxynitrite as shown in Figure 3. Once formed, peroxynitrite uncouples eNOS via oxidizing and depleting either the eNOS substrate L-arginine, the cofactor tetrahydrobiopterin, or both. Uncoupled eNOS produces more superoxide than NO, resulting in reduced NO levels and the formation of increasing amounts of peroxynitrite in the same compartment. This uncoupling of eNOS via peroxynitrite may lead to endothelial cell activation and enhanced expression of ICAM-1, thus compromising BBB integrity (Figure 5A-B) and causing a neuroinflammatory secondary injury (Figure 6) to the neurovascular unit [41,70]. Inflammation-induced monocyte-endothelial cell interaction and up regulation of ICAM-1 have been reported to be inhibited by GSNO-mediated S-nitrosylation of NF-κB [17], indicating that GSNO invokes its anti-inflammatory effect through S-nitrosylation. eNOS is also reported to be dynamically regulated by S-nitrosylation at Cys^101^, resulting in inhibition of eNOS activity [81,82]. Therefore, GSNO-mediated reduced levels of peroxynitrite formation (Figures 1, 2, 3) and the consequent BBB protection (Figure 5) may have resulted from the inhibition of aberrant eNOS by S-nitrosylation. The protective effect of S-nitrosylation signaling is well documented in the cardiovascular system [83]. A depletion of circulating nitroso/nitrosyl species in plasma has been observed in patients with endothelial dysfunction [84]. Deficient S-nitrosylation is considered a general mechanism in neurodegenerative diseases [27,85-87]. GSNO also inhibits platelet activation [22,23] and protects BBB integrity and epithelial permeability [2,88]. Like endothelial cells, neuronal cells also produced peroxynitrite (Figure 2B), which may induce cell death, thus compromising neurorepair. GSNO treatment of TBI also reduced these neuronal peroxynitrite levels, likely via nitrosylating and inhibiting neuronal NOS activity, thereby stimulating neurorepair mechanisms. Neuronal NOS activity has been shown to be down regulated by GSNO via nitrosylation of the NMDA receptor [89].

*In vivo*, the action of GSNO is modulated mainly by nitrosylation/transnitrosylation [7,8,18]. Via nitrosylation, GSNO exerts its anti-inflammatory action mainly through inhibition of NF-κB, TNF-α, and iNOS/neuronal NOS [3,16,90,91], its antiapoptotic action through inhibition of caspase-3 activity [19], and its antioxidant activity through down regulation of peroxynitrite [20,21] and up regulation of GSH [4]. Another target of GSNO might be the inhibition of superoxide production by nitrosylation of NADPH oxidase. Nitrosylation of p47phox of NADPH oxidase has been reported to suppress superoxide production in human endothelial cells [92]. Superoxide is implicated in the reduction of GSNO because NO has higher reactivity with superoxide than GSH, a substrate of GSNO biosynthesis. Reduced levels of GSNO may also result from its decomposition (denitrosylation) by the enhanced expression of GSNO reductase [8]. The expression of GSNO reductase is increased during inflammation [8]. GSNO reductase is the only known enzyme that degrades GSNO without releasing NO. Therefore, an exogenous supplementation of GSNO under neuroinflammatory conditions may reduce the oxidative burden (Figures 4, 8) and protect BBB integrity (Figure 5A-B), resulting in reduced edema (Figure 5C) and decreased brain damage (Figures 7, 9). Moreover, GSNO/hemoglobin homeostasis is required in the circulation to maintain GSNO-based S-nitrosylation mechanisms. Enhancing S-nitrosylation by ethyl nitrite, a nitrosylating agent of hemoglobin, has improved outcomes in mice with subarachnoid hemorrhage [93].

Our previous study on neurovascular protection by S-nitrosylating agents in TBI and stroke shows that GSNO down regulated the expression of inflammatory mediators ICAM-1, ED-1, iNOS, MMP-9, protected the BBB, and improved neurological functions [2,3]. In this study, we observed that a long-term post-TBI treatment with GSNO not only improved tissue structure (Figures 7, 9), reduced the expression of ICAM-1 (Figure 6), and restored myelin/axon loss (Figure 9) but also increased synaptogenesis (increased expression of synaptophysin; Figure 10) and stimulated the expression of neurotrophic factors (enhanced expression of BDNF and TrkB; Figure 10). Neurorepair following brain injury is facilitated by synaptophysin and BDNF [94,95]. Synaptophysin is a protein involved in the biogenesis of synaptic vesicles and budding. NO can influence plastic changes in specific brain regions and can change the expression of synaptophysin [95]. Because NO is converted to peroxynitrite in an oxidative environment such as that which occurs in TBI, it is difficult to dissect the effects on neurotrophins of NO/peroxynitrite vs. nitrosylation. However, our data showing enhanced expression of synaptophysin, BDNF and TrkB in the GSNO brain compared to the TBI (Figure 10) indicate that nitrosylation accelerates the neurorepair process. Preservation of myelin and axons in the GSNO group (Figure 9) further supports GSNO-mediated mechanisms of neurorecovery.

The therapeutic potential of GSNO is also supported by increased levels of GSH and TGSH in the GSNO brain following TBI (Figure 8). Higher levels of TGSH in the GSNO group compared to TBI and SIN-1 are likely due to increased GSSG, which may be converted back to GSH by GSH reductase. GSNO may also increase GSH indirectly via neutralizing ROS. A similar observation after GSNO treatment in our IR study [4] indicates that GSNO's infarct-reducing effect (Figure 7) may be dependent on its antioxidant activity.

Because clinically proven TBI therapy is not currently available and GSNO shows clinically relevant therapeutic potential in our animal model of TBI, a further investigation is warranted to test GSNO's efficacy in human TBI. GSNO occurs naturally in the human body and is safe, as its exogenous administration in humans for other indications was not associated with toxicity or side effects [24,26,96].

## Conclusion

GSNO reduces peroxynitrite and neuroinflammation, increases NO and GSH, and protects the integrity of the BBB and the neurovascular unit following TBI. Furthermore, it stimulates the mechanisms of the neurorepair in the injured animals. Therefore, GSNO has therapeutic potential to be investigated in human TBI.

## List of Abbreviations

BBB: blood brain barrier; BDNF: brain-derived neurotrophic factor; CBF: cerebral blood flow; CCI: controlled cortical impact; CNS: central nervous system; EB: Evan's blue; eNOS: endothelial nitric oxide synthase; GSNO: S-nitrosoglutathione; H&E: hematoxylin and eosin; HR: heart rate; ICAM: intracellular adhesion molecule; IHC: immunohistochemistry; iNOS: inducible nitric oxide synthase; IR: ischemia-reperfusion; LFB: luxol fast blue; MABP: mean arterial blood pressure; MMP: matrix metalloproteinase; mRNA: messenger ribonucleic acid; NF-κB: nuclear factor kappa B; NO: nitric oxide; NOS: nitric oxide synthase; RNS: reactive nitrogen species; ROS: reactive oxygen species; RTPCR: real-time polymerase chain reaction; Sham: sham-operated animals; SCI: spinal cord injury; SIN-1: 3-Morpholino-sydnonimine; TBARS: thiobarbituric acid reactive substances; TBI: traumatic brain injury; TNF: tumor necrosis factor; TrkB: tropomyosin-related kinase B; TTC: 2,3,5-triphenyltetrazolium chloride.

## Competing interests

The authors declare that they have no competing interests.

## Authors' contributions

This study is based on an original idea of MK and IS. MK wrote the manuscript. HS, TSD, AS, MK, AGG, and YI carried out animal and biochemical studies. MK, AKS, AS, and HS critically examined histochemical studies. All authors have approved the manuscript.
